# Draft Genome Sequence of *Colletotrichum sublineola*, a Destructive Pathogen of Cultivated Sorghum

**DOI:** 10.1128/genomeA.00540-14

**Published:** 2014-06-12

**Authors:** Riccardo Baroncelli, José María Sanz-Martín, Gabriel E. Rech, Serenella A. Sukno, Michael R. Thon

**Affiliations:** Instituto Hispano-Luso de Investigaciones Agrarias (CIALE), Departamento de Microbiología y Genética, Universidad de Salamanca, Salamanca, Spain

## Abstract

*Colletotrichum sublineola* is a filamentous fungus that causes anthracnose disease on sorghum. We report a draft whole-genome shotgun sequence and gene annotation of the nuclear genome of this fungus using Illumina sequencing.

## GENOME ANNOUNCEMENT

Sorghum (*Sorghum bicolor*) is an important cultivated crop and is used as a staple food, as animal fodder, and as a biofuels feedstock. One of the most important diseases that affects sorghum crops is sorghum anthracnose, caused by the ascomycete fungus *Colletotrichum sublineola*, which can cause a reduction in grain size as well as yield losses of more than 50% under epidemic conditions (1). *Colletotrichum sublineola* is also closely related to *C. graminicola* (2), a causal agent of maize anthracnose, and is therefore useful for comparative genomics of the two species to uncover the evolutionary mechanisms of speciation, host specificity, and pathogenicity.

*Colletotrichum sublineola* strain TX430BB was isolated from sorghum in College Station, TX, USA (3). Total genomic DNA was purified using the method of Baek and Kenerley (4) and sequenced using 100-bp paired-end reads on an Illumina HiSeq 2000, and the sequence reads (4.32 Gbp; average coverage, 91.93×) were assembled using Velvet version 1.2.07 (5). The draft genome of *C. sublineola* consists of 1,625 sequence scaffolds with a total length of 46.75 Mbp (*N*_50_=70,717 bp, and *N*_90_=13,454 bp), 52.70% G+C content, and a maximum scaffold size of 423,147 bp. The mitochondrial genome was identified by performing BLAST searches of the contigs in a database of mitochondrial genomes of other fungi, resulting in the identification and removal of 6 contigs. The completeness of the assembly was assessed using CEGMA v2.4 (6), which estimated the genome sequence to be 98.39% complete. The nuclear genome was annotated using the MAKER pipeline (7). Overall, 12,699 protein-coding gene models were predicted in the nuclear genome.

Using WoLF PSORT (8) we identified 1,820 proteins that are predicted to be extracellular (14.33% of the proteome). Based on BLAST searches (*e*-value threshold of 1*e*−3) of the extracellular proteins, 168 (9.23% of the secretome) do not have any sequence similarity to proteins in *C. graminicola*, and of those 168 proteins, 70 (3.85% of the secretome) do not have any sequence similarity to proteins in other *Colletotrichum* species (2, 9–11). However, only 60 secreted proteins are unique to *C. sublineola* when compared to the nr database with BLAST. These species-specific extracellular proteins may be effectors, proteins that have important roles in modulating the plant’s immune system and in host specialization.

In this study we present a draft genome sequence from a member of *C. sublineola*, a destructive pathogen of cultivated sorghum. The sequence represents a new resource that will be useful for further research into the biology, ecology, and evolution of this key pathogen.

### Nucleotide sequence accession numbers.

This whole-genome shotgun sequencing project has been deposited at GenBank under the accession no. JMSE00000000. The version described in this paper is JMSE00000000.1.
